# Hospital-based HIV/HSV-2 seroprevalence among male patients with anal disease in Korea: cross sectional study

**DOI:** 10.1186/1471-2334-14-34

**Published:** 2014-01-20

**Authors:** Jin-Sook Wang, Do Yeon Hwang, Hye-Kyung Yu, Sung Soon Kim, Jong Kyun Lee, Mee-Kyung Kee

**Affiliations:** 1 Division of AIDS, Center for Immunology and Pathology, Korea Centers for Diseases Control and Prevention, Osong Health Technology Adminstration Complex, 187 Osongsaengmyeong2(i)-ro, Cheongwon-gun, Chungcheongbukdo 363-951, Korea; 2 Division of Biobank for Health Sciences, Korea Centers for Diseases Control and Prevention, Osong, Chungcheongbuk-do, Korea; 3 Department of Surgery, Song Do Medical center, Seoul, Korea

**Keywords:** HIV/HSV-2, HIV seroprevalence, HSV-2 seroprevalence, Anal disease, Age group

## Abstract

**Background:**

This study aimed to identify the characteristics of HIV and herpes simplex virus (HSV)-2 seroprevalence in male patients with anal disease.

**Methods:**

HIV seroprevalence was estimated for different age groups of male patients with anal disease who were treated at Songdo colorectal hospital in Korea between 2001 and 2011. HIV seroprevalence of patients with anal disease was compared with that of patients with nonanal disease for each year from 2007 to 2011. HSV-2 antibody tests were conducted on 2,038 HIV-tested male patients with anal disease in 2009.

**Results:**

For 11 years from 2001, HIV seroprevalence differed significantly by age group (*P* < 0.001) and was highest in the group aged <20 years. From 2007 to 2011, HIV seroprevalence in patients with anal disease was 7.6/10,000–13.3/10,000 and that in patients with nonanal disease was 0–0.9/10,000. HSV-2 seroprevalence among patients with anal disease was 24.0%, and only one patient with HIV and HSV-2 was observed.

**Conclusions:**

HIV seroprevalencein male patients with anal disease was significantly higher than that for other diseases. HSV-2 seroprevalence was similar to that in the general male population. Implementation of the current HIV surveillance system for male patients at colorectal hospitals is necessary to help prevent HIV transmission.

## Background

When AIDS was first reported in 1981, it seemed to be a new disease that affected only homosexual men. However, it was discovered that the disease spread to the general population through drug users, hemophilia patients, children infected by their mothers, and sexual partners of individuals with HIV through blood or sexual contact [1]. Since 1985, when the first case of HIV infection was identified in Korea, the number of HIV infections has continued to increase, with the cumulative total reaching 9,406 in 2012. The ratio of males to females among reported HIV cases is approximately 11:1. Overall, 99% of HIV infections is caused by sexual contact, and the proportion of cases attributed to male-to-male sexual contact is about 40% [2]. Almost 70% of all newly identified cases is identified during hospital care [3]; thus, it was of interest to identify the type of medical procedures and treatment during which the highest number of new HIV diagnoses was made. According to Miles et al. and Wexner et al., 5.9–34% of the HIV-infected population has anal disorders, which are also the most frequent cause for surgery in this group [4,5]. Among men who have sex with men attending a sexually transmitted infection (STI) clinic, 30.8% had dysuria, pus, and urethral ulceration, and 37.1% had pus, bleeding, ulceration, and/or pain in the rectum [6]. A study of HIV infection and anal diseases in Korea found that most HIV infections detected at colorectal hospitals were in men, most of whom were homosexual [7]. Currently, there are more males than females HIV patients and the predominant route of transmission is homosexual contact in Korea.

Genital ulcers have been associated with increased risk factors for HIV infection, and genital herpes is usually caused by herpes simplex virus (HSV)-2 [8]. Our previous study had compared HSV-2 seroprevalence in HIV-infected males with that in the general male population in Korea, which was two or three times higher among HIV-infected males [9]. HSV-2 seroprevalence had a tendency to increase with age and HIV seroprevalence had a different characteristic by age in most countries including Korea [9-11].

Given these epidemiological characteristics of HIV infection in Korea, we aimed to estimate HIV seroprevalence and trends among men suffering from anal disease. We also aimed to identify the seroprevalence of these sexually transmitted viruses by measure of HSV-2 infection, a virus that is spread sexually.

## Methods

### Subjects

HIV seroprevalence in male patients who were treated for anal disease at Songdo (S) colorectal hospital from 2001 to 2011 was estimated for different age groups. S colorectal hospital is a representative large hospital specializing in the field of anal diseases in Seoul, Korea. HIV seroprevalence in male patients with anal disease was compared with that in those who presented for other reasons (including a health checkup) calculated for each year from 2007 to 2011. To understand HIV and/or HSV-2 seroprevalence, HSV-2 tests were conducted on 2,038 men who received treatment for anal diseases at S colorectal hospital. The HSV-2 samples randomly selected among 26,173 male patients with anal disease tested for HIV in 2009. Ethics approval was obtained from KCDC Institutional Review Board (IRB) Ethics Committee.

### HIV and HSV-2 antibody tests

HIV screening was performed using Architect HIV AG/AB Combo Kit (Abbott Laboratories, Abbot Park, IL, USA) to determine the presence of HIV antibody and/or antigen. Patient samples that tested positive in HIV screening were subjected to an additional HIV western blot test (HIV BLOT 2.2 Western Blot Assay, MP Diagnostics, Asia Pacific Pte Ltd., Singapore) at an HIV confirmatory testing institute to confirm HIV infection. HSV-2 antibody tests were performed with the HerpeSelect 2 ELISA IgG Kit (Focus Diagnostics, Cypress, CA, USA). HSV-2 results were reported as an index value relative to the cut-off calibrator. An index value >1.10 was presumptive for the presence of IgG antibodies to HSV-2.

### Statistical analysis

HIV seroprevalence was defined as the number of confirmed HIV cases per 10,000 HIV-tested people over a 1-year period. HSV-2 seroprevalence was defined as the proportion of HSV-2-positive people among the total number of people tested. Patients with any type of anal disease were grouped together as the anal disease patients, and patients who visited the hospital for a health checkup or for another intestinal disorder were categorized as the nonanal disease patients. Trend tests were conducted to determine how HIV seroprevalence varied from year to year in the anal and nonanal disease patients, using SAS version 9.3. χ^2^ tests were used to compare HIV seroprevalence in patients with anal disease and nonanal disease, and HIV and HSV-2 seroprevalence in different age groups of patients with anal disease, with a *95%* confidence level.

## Results

### Status of HIV tests and trends in HIV seroprevalence

**Figure 1:**
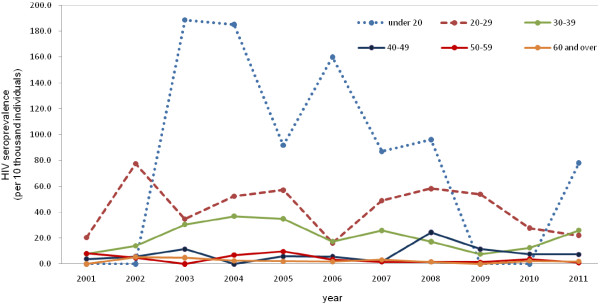
**Trends in HIV seroprevalence by age among male patients with anal diseases, 2001–2011.** Annual HIV seroprevalence was 6.3 to 16.2 per 10,000 individuals.

S colorectal hospital administered HIV tests to 17,593–30,225 male patients with anal disease every year from 2001 to 2011. Approximately 50% of total HIV tests were performed in patients aged ≥50 years. Overall, 255 HIV-infected males were founded during the same period (data not shown). As shown in Figure 1, annual HIV seroprevalence among male patients with anal disease during the study period was 6.3–16.2 per 10,000. The trend revealed that HIV seroprevalence was highest in the group aged <20 years, followed by 20–29, 30–39, 40–49 and ≥50 years (*P* < 0.0001). Over 11 years, 1,254 teenagers (approximately 0.5% of the total) were treated for anal disorders and among these, 10 were diagnosed with HIV (data not shown). The trend in HIV seroprevalence over time did not show any specific pattern in any of the age groups: under 20 (*P* = 0.5982), 20–29 (*P* = 0.3675), 30–39 (*P* = 0.7476), 40–49 (*P* = 0.1336), 50–59 (*P* = 0.0815), and ≥60 (*P* = 0.3709) years.

As shown in Table 1, for the trend in HIV seroprevalence among male patients with anal or nonanal disease, 119 were found to be positive for HIV from 2007 to 2011. One hundred and sixteen male patients with anal disease were confirmed to have HIV (97% of total confirmed infections). HIV seroprevalence in patients with anal disease ranged from >10/10,000 in 2007 to 7.6/10,000 in 2011. Among patients with nonanal disease, three were found to have HIV with an estimated seroprevalence of 0.5/10,000 in 2008 and 0.9/10,000 in 2010.

**Table 1:** Trend in HIV seroprevalence among male patients with anal disease or with nonanal disease, 2007 - 2011

**Year**	**With anal diseases**			**With nonanal diseases**		
	**N (%)**^**a**^	**HIV +**^**b**^	**HIV seroprevalence per 10,000**	**N (%)**^**a**^	**HIV +**^**b**^	**HIV seroprevalence per 10,000**
2007	23,861 (58.0)	24	10.1	17,274 (42.0)	0	0
2008	22,581 (54.3)	30	13.3	18,986 (45.7)	1	0.5
2009	26,173 (53.2)	20	7.6	23,000 (46.8)	0	0
2010	27,552 (56.3)	19	6.9	21,378 (43.7)	2	0.9
2011	30,225 (56.4)	23	7.6	23,382 (43.6)	0	0

HIV prevalence was defined as the number of confirmed HIV cases per 10,000 HIV-tested people over a 1-year period.

Nonanal diseases: patients who presented at the hospital with a nonanal disease or for a health checkup.

^a^N (%): the number of individuals tested for HIV (the proportion of patients with anal disease or nonanal disease).

^b^HIV+: the number of HIV-infected individuals.

### HIV and/or HSV-2 seroprevalence among patients with anal disease

As shown in Table 2, of the 2,038 male patients with anal disease, 1.8% were aged <20 years, 11.0% were 20–29 years, 20% were ≥30 years. Five men (25/10,000) were diagnosed with HIV, three of whom were in their 20s, and one each in the 30–39- and 40–49-year age groups. The total HSV-2 seroprevalence was 24.0%: 5.4% for men aged <20 years, 8.0% for those aged 20–29 years, and 11.7% for those aged 30–39 years, with the rate increasing with age (*P* < 0.0001). Infection with HIV and HSV-2 was found in only one patient (4/10,000) of the total number tested, who was in his 30s.

**Table 2:** HIV seroprevalence and HSV-2 seroprevalence by age among male patients with anal disease, 2009

**Age**	**No. (%)**	**HIV +**^**a**^	**HIV seroprevalence per 10000**	**HSV-2 +**^**b**^	**HSV-2 seroprevalence (%)**
Total	2,038 (100)	5	24.5	489	24.0
< 20	37 (1.8)	0	0	2	5.4
20–29	224 (11.0)	3	134	18	8.0
30–39	446 (21.9)	1	22	52	11.7
40–49	398 (19.5)	1	25	85	21.4
50–59	477 (23.4)	0	0	166	34.8
≥ 60	456 (22.4)	0	0	166	36.4

HIV seroprevalence was defined as the number of confirmed HIV cases per 10,000 of HIV-tested people.

HSV-2 seroprevalence was defined as the proportion of HSV-2-positive people. The 2,038 HSV-2 samples randomly selected among 26,173 male patients with anal disease tested for HIV in 2009.

^a^HIV+: the number of HIV-infected individuals.

^b^HSV2: the number of HSV-2-infected individuals.

## Discussion

In the present study, HIV seroprevalence among male patients with anal disease was between 6.3/10000 and 16.2/10000, which was nearly 30 times higher than that of male patients with nonanal disease at a colorectal hospital. This indicates a strong association between anal disease and HIV infection in male patients. Over the past 11 years (Figure 1), HIV seroprevalence in male patients with anal disease has decreased, although the status of HSV-2 seroprevalencehas remained at a level similar to that in the general male population.

According to the Joint United Nations Programme on HIV/AIDS (UNAIDS), HIV seroprevalence in Korea is at a low epidemic level of between 1/10,000 and 4/10,000 [12,13]. However, the number of HIV-infected individuals in Korea has continued to rise and isexpected to increase further, for two main reasons. First, the HIV epidemic pattern in Korea is similar to that observed in the early 1980s when HIV became a global pandemic. More specifically, the epidemic pattern of HIV in Korea closely follows the pattern of the early epidemiological stages of the virus when HIV was spread mainly through sexual contact, especially between men [14]. Second, in many of the recently identified cases, the infections were discovered long after onset. This means that the infected individuals may have unknowingly transmitted the virus to other people [3]. Moreover, an increasing number of HIV cases are detected during health checkups and treatment of other conditions [13]. Therefore, the role of private health-care institutions has become important in both HIV treatment and diagnosis.

In a prior study that examined HIV seroprevalence during 2002 and 2007, based on data collected from colorectal hospitals across the country, HIV seroprevalence was between 2.2/10,000 and 3.9/10,000, which was about two or three times higher than that of all hospital patients [13,15]. This study analyzed the characteristics of HIV seroprevalence among patients in a colorectal hospital according to variables including sex, age and presence of anal disease. The results showed that most of the cases of HIV were in patients who also had anal disease. Overall, HIV seroprevalence in patients with anal disease was ≈ 10/10000. In Korea, HIV seroprevalence was highest among sexual partners of HIV-infected individuals (>1,000/10,000), followed by people who applied voluntarily for HIV testing because they wanted to know their status (>20/10,000), and people who underwent additional HIV tests when their doctor suspected HIV infection during treatment (>10/10,000) [11,16]. This study showed that male patients with anal disease were similar to those referred by their doctor and were assumed to be a high-risk group.

In addition, male patients in whom HIV infection was detected at the colorectal hospital tended to be younger than the general HIV-infected male population, and HIV seroprevalence increased significantly with younger age. For specific identification of high HIV seroprevalence in the group aged <20 years, we divided into groups aged <10 years, 10–14 years and 15–19 years. From 2001 to 2011, 1,061 male patients aged 15–19 years were treated and underwent HIV testing at the hospital. Ten HIV infections were found in the group aged 15–19 years.

In Korea, the HIV-infected young men aged 15–19 years accounted for 2.4% of the total number of HIV-infected men as of 2011. Sixty percent of these were infected through homosexual contacts, which is higher than the overall percentage infected through homosexual contact (43%) [17]. In the present study, male patients aged 15–19 years comprised 3.7% of the total number of male patients with HIV and anal disease. This produced a higher annual HIV seroprevalence than for any other group nationwide in Korea.

A survey of male sexual behavior in Korea showed that sexual activity began around age 16 years [18], and only 10–20% used condoms [19]. A survey of homosexual behavior in Korea showed that 60% of participants had a homosexual feeling before age 18 years [20]. It is expected that the group aged 15–19 years is a group at high risk for STI.

Approximately 70% of HIV-infected individuals in the present study contracted the virus through homosexual contact, according to the HIV database of KCDC. In Korea, HIV infection was detected in many people who visited HIV testing and consulting centers for homosexual people (HIV-positive rate of 5.5%) [21]. However, the proportion of homosexual men who tested for HIV over the past year in Korea was lower than that in other countries including Japan, China and the United States [22]. Moreover, nearly half of all homosexual men in Korea said that they had never tested for HIV [20]. Therefore, the present study shows that ensuring that colorectal surgeons can easily identify lesions associated with HIV infection in male patients with anal disease is important for preventing misdiagnosis or delayed diagnosis of HIV infection and stopping its spread.

HSV-2 can often cause acute infection or remain inactive until it is triggered by a decline in the immune system. HSV-2 is the cause of >90% of anorectal infections and frequently the source of proctitis in homosexual men [23]. In homosexual men living in San Francisco, the seroprevalence of HSV-2, HIV and HSV-2/HIV was 26.1%, 18.6% and 12.0%, respectively [24]. In male infected with HIV by sexual contacts in Korea in 2003, HSV-2 seroprevalence (23.9–69.2%) was higher than that in the general male population (6.8–26.0%) [9]. In the present study, 20% of HIV-infected individuals were found to have HSV-2. Moreover, HSV-2 seroprevalence in male patients with anal disease was similar to that in the general male population.

The present study had the following limitations. First, there is limited scope to generalize the findings of the study, because the estimates of HIV seroprevalence were based on data obtained from a single colorectal hospital located in Seoul. However, the study did show the trends, status, and characteristics of HIV seroprevalence among male patients with anal disease. Second, although the estimated HSV-2 seroprevalence in male patients with anal disease was flawed because the results were based on data from a single hospital during a limited period, the estimate is important because it is believed to be the first such study in Korea. Finally, we don’t have data on sexual behavior to find out the reason of high HIV seroprevalence among male patients with anal disease. And, HSV-2 seroprevalence was low in male patients with anal disease, which differed from other studies [25,26]. It will be necessary to establish the relationship between the study results and the epidemiological characteristics of the study subjects in Korea.

## Conclusions

The HIV seroprevalence of male patients with anal disease was significantly higher than that in patients with other diseases. The high HIV seroprevalence in patients in a colorectal hospital suggests the need to develop effective prevention programs for sexually transmitted infections, with a focus on hospitals specializing in anal diseases.

### Ethics statement

Ethics approval was obtained from KCDC Institutional Review Board (IRB) Ethics Committee.

## Abbreviations

(STI): Sexual transmitted infection; (HSV-2): Herpes simplex virus type 2; (KCDC): Korea centers for diseases control and prevention; (S colorectal hospital): Songdo colorectal hospital.

## Competing interest

The authors declare that they have no competing interests.

## Authors’ contributions

J-SW, DYH and SSK designed and conceived the idea for the study and M-KK supervised all aspects of its implementation. J-SW wrote the first draft of the manuscript. DYH and JKL contributed to the collection and interpretation of raw data. M-KK and H-KY completed all data analyses and SSK coordinated funding for the project. All authors read and approved the final version of the manuscript as submitted to *BMC Infectious Diseases*.

## Pre-publication history

The pre-publication history for this paper can be accessed here:

http://www.biomedcentral.com/1471-2334/14/34/prepub
